# Innate immune sensors of inflammasomes and PANoptosomes: structural–mechanistic insights and therapeutic implications

**DOI:** 10.1038/s41422-026-01287-9

**Published:** 2026-09-04

**Authors:** Saurabh Upadhyay, Raghavendra Nagampalli, Sara Resende, Thirumala-Devi Kanneganti

**Affiliations:** Department of Immunology, St. Jude Children’s Research Hospital, Memphis, TN USA

**Keywords:** Molecular biology, Biological techniques

## Abstract

Innate immunity provides a critical first line of defense against pathogens and homeostatic perturbations. Pattern recognition receptors detect these disruptions and initiate immune responses through multi-protein complex formation to drive inflammatory signaling and cell death pathways. Key cytosolic complexes formed by these sensors include inflammasomes and PANoptosomes. Inflammasomes induce caspase-1 activation and the subsequent maturation of interleukin (IL)-1β and IL-18, and they can act as integral components of larger PANoptosomes, whose formation and functions have been defined by genetic, biochemical, and single-cell imaging evidence. PANoptosomes induce lytic, inflammatory cell death (PANoptosis) and promote the release of damage-associated molecular patterns (DAMPs) and cytokines beyond IL-1β and IL-18, including TNF, IFNs, IL-6, and others. Given their critical functions in driving cell death and the release of cytokines and DAMPs, dysregulation of innate immune sensors is associated with a wide range of diseases, including infections, autoinflammatory syndromes, cardiovascular disorders, neurodegeneration, metabolic conditions, and cancer. Therefore, understanding innate immune sensors and how they assemble inflammasomes and PANoptosomes to drive cell death is critical for identifying therapeutic strategies. In this review, we discuss innate immune sensors that form inflammasomes and PANoptosomes, such as NLRP1, NLRP3, NLRC4, AIM2, Pyrin, and others. We highlight recent structural and mechanistic insights into these sensors, along with emerging structural studies of inflammasome assemblies and the biochemical and functional evidence supporting the formation of PANoptosomes. Given the physiological relevance of innate immune sensors and the complexes they form across the disease spectrum, an improved understanding of their structure–function relationships will be critical for informing therapeutic strategies that target these molecules, their associated complexes, and their physiological functions.

## Introduction

The innate immune system serves as the body’s first line of defense against pathogens and sterile insults through pattern recognition receptors (PRRs). These receptors detect pathogen- and damage-associated molecular patterns (PAMPs and DAMPs) as well as homeostatic perturbations.^1,2^ Upon sensing their cognate ligands, PRRs initiate immune pathways, including inflammatory signaling, cytokine production, and cell death. These responses are essential for host defense and the maintenance of homeostasis.^3,4^ However, excessive or dysregulated PRR activation can lead to pathological inflammation, cytokine storm, and additional DAMP release, ultimately resulting in severe tissue damage, organ failure, and lethality.^5^ Therefore, gaining an in-depth understanding of PRR structure–function relationships and defining their roles in disease are essential for developing therapies targeting inflammation and disease.

Among the PRRs, certain cytosolic sensors, including NLRP1, NLRP3, NLRC4, along with AIM2 and Pyrin, can detect pathogens or sterile insults and form inflammasomes.^6–14^ Inflammasomes are cytosolic multi-protein complexes that, upon sensor activation, lead to caspase-1 autoproteolytic cleavage and activation. Active caspase-1 then cleaves its downstream substrates, such as the cytokines interleukin (IL)-1β and IL-18 and pore-forming molecule gasdermin D (GSDMD).^15–19^ Caspase-1-dependent cell death is known as pyroptosis.^20^ Inflammasome activation can proceed through both canonical^6,7,8^ and non-canonical^21,22^ pathways, which differ in their activation mechanisms.^23^

Beyond their role in driving pyroptosis, inflammasomes can act as integral components of PANoptosomes, which drive a form of cell death called PANoptosis. PANoptosis is defined as an innate immune, lytic, inflammatory cell death pathway initiated by innate immune sensors and driven by caspases and receptor-interacting protein kinases (RIPKs) through PANoptosome complexes.^24–30^

PRRs known to form PANoptosomes include ZBP1, AIM2, RIPK1, NLRP3, NLRC5, and NLRP12.^24–30^ Phenotypically, PANoptosis is characterized by the activation of molecules classically associated with pyroptosis, apoptosis, and necroptosis, including caspase-1, caspase-3, caspase-7, caspase-8, RIPK1, and RIPK3. However, PANoptosis is distinguished from these pathways by the formation of PANoptosome complexes, which have been identified in individual cells through extensive single-cell imaging analyses,^24,28,30–34^ and by the genetic essentiality of key molecules. For example, while caspase-1 is genetically required for pyroptosis, PANoptotic cell death proceeds even in caspase-1-deficient cells.^26,27,30,32,35–39^ Furthermore, PANoptosis proceeds in cells deficient in RIPK3 (a core necroptotic kinase) or in cells expressing an enzymatically inactive form of caspase-8, which abolishes extrinsic apoptosis and caspase-8-dependent secondary necrosis.^26,27,30,32,35–38^ These results suggest that PANoptotic cell death is not dependent on caspase-1, RIPK3, or caspase-8 enzymatic activity alone. Instead, PANoptosis can be blocked only with combined deletions of these molecules, or by deletion of the upstream sensor.^26,27,30,32,35–38^ To date, the ZBP1-, AIM2-, RIPK1-, NLRP3-, NLRP1-, and NLRC5- and NLRP12-PANoptosomes have been molecularly characterized, with extensive genetic, molecular, and biochemical evidence supporting their formation and functions.^24–30,39–46^

Inflammasomes and PANoptosomes share similar upstream regulatory mechanisms that drive their activation. Both are initiated by innate immune sensors that respond to microbial or sterile stimuli and rely on adaptor engagement and caspase recruitment to execute downstream responses. In the case of NLRP3, NLRP1, and AIM2, the sensors can initiate both inflammasome and PANoptosome assembly, depending on the trigger.^7–9,13,14,24,25,28,30,39^ This suggests that there are trigger-dependent regulatory events that distinguish inflammasome and PANoptosome activation. In general, inflammasomes are triggered by a single PAMP or DAMP, while PANoptosomes are triggered by physiologically relevant homeostatic alterations, cytokines, and PAMPs/DAMPs, such as those from whole pathogens (e.g., *Francisella*, influenza),^24,25^ metabolic stressors (e.g., NAD^+^ depletion),^28,47^ and cytokine combinations during cytokine storm (e.g., TNF plus IFN-γ).^35^ Although understanding the activating mechanisms that distinguish inflammasome and PANoptosome formation remains an active area of study, emerging evidence suggests that transcriptional regulators such as interferon regulatory factor 1 (IRF1) play a prominent role in licensing PANoptosome assembly.^48^ Further investigation will be required to define additional molecular features that distinguish inflammasome and PANoptosome activation.

Despite the compelling genetic, biochemical, and imaging evidence supporting the formation of both inflammasomes and PANoptosomes, atomic-resolution structures of these fully assembled complexes remain unavailable. Advances in structural biology, such as cryo-electron microscopy (cryo-EM), cryogenic fluorescence-guided focused ion beam (cryo-FIB) milling, and electron cryo-tomography (cryo-ET), have provided insights into sensor-level structures and partial inflammasome architectures, but complete higher-order inflammasome and PANoptosome assemblies are structurally unresolved. In this review, we provide a comprehensive structural overview of key innate immune sensors involved in forming inflammasomes and PANoptosomes. We describe the structural features of the sensors, as well as insights into how these structural features inform complex formation and activation mechanisms. To do this, we focus on the different levels of structural information that are available: (i) experimentally solved sensor-level structures, (ii) partially resolved inflammasome architectures supported by a combination of structural and modeling approaches, and (iii) PANoptosome assemblies, for which complex formation is conclusively supported by genetic, biochemical, and imaging data but whose higher-order architectures remain structurally unresolved (Table 1). We also highlight the involvement of the sensors in various diseases, discuss how they contribute to disease progression, and review their therapeutic potential. As structural insights continue to expand, this review aims to lay a foundation for future studies and to compile the existing structure-function data that can inform therapeutic strategies targeting diseases associated with innate immune sensors and inflammatory cell death complex formation.

**Table 1:** Comparison of inflammasomes and PANoptosomes: sensors, core components, and structural evidence.

	Inflammasomes	PANoptosomes
Sensors	NLRP1, NLRP3, NLRC4, AIM2, Pyrin	ZBP1, NLRP3, NLRP1, AIM2, RIPK1, NLRP12, NLRC5
Components	Adaptors (ASC) Effectors (Caspase-1)	Adaptors (e.g., ASC, FADD) Effectors (Caspase-1, Caspase-8, RIPK1/RIPK3)
Current structural evidence	**Sensor-level structures:** NLRP1^54,55^ NLRP3^90,95^ NLRC4^179,297^ AIM2^196,197^ Pyrin^237^ **Inflammasome architectures:** Partial assemblies — ASC filaments^96,280^ CASP1-CARD filaments^280^ NLRP3 cages^90,96^ NLRC4 multimers^298^ No complete atomic-resolution structures for fully assembled inflammasomes	**Sensor-level structures:** NLRP1^54,55^ NLRP3^90,95^ AIM2^196,197^ **Domain-level sensor structures**: ZBP1^299,300^ RIPK1^301–303^ NLRP12^304,305^ NLRC5^306^ **PANoptosome architectures:** Inferred from inflammasome partial assemblies — ASC filaments^96,280^ CASP1-CARD filaments^280^ NLRP3 cages^90,96^ No complete atomic-resolution structures for fully assembled PANoptosomes

## Nlrp1

### Structural features of NLRP1

**Fig. 1:**
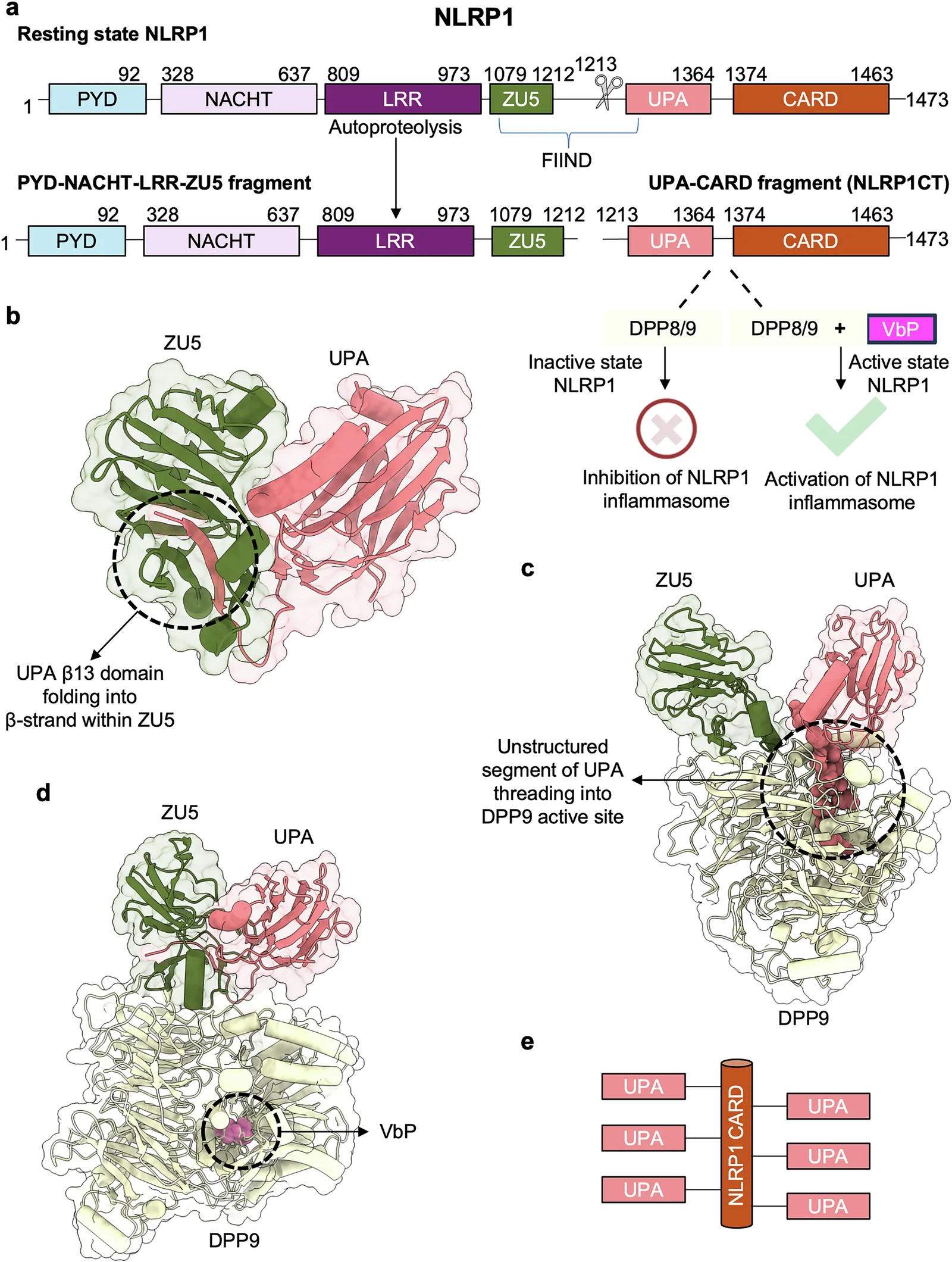
Structural insights into NLRP1 activation. **a** Domain architecture of NLRP1 and schematic illustration of NLRP1 activation and regulation. The FIIND proteolysis site that releases the N-terminal fragment for proteasomal degradation is shown. DPP9 inhibits NLRP1 activation by preventing UPA-CARD oligomerization, while VbP activates NLRP1 by blocking the inhibitory effect of DPP9. Residue numbers for domain boundaries are indicated based on the human protein sequence. **b** The structure of ZU5 and the UPA region within the FIIND showing the intramolecular interaction (PDB: 7CRV). The UPA domain adopting a β-sheet conformation within ZU5 is also shown. **c** Cryo-EM structure of the NLRP1–DPP9 ternary complex showing insertion of the N-terminus of the NLRP1 UPA-CARD fragment into the active-site tunnel of DPP9, as labeled (PDB: 6X6A). **d** The structure of DPP9 bound to the small-molecule inhibitor VbP (PDB: 6X6C). VbP activates NLRP1 by displacing the N-terminal segment of the UPA domain from the DPP9 active site, which releases the UPA-CARD fragment and initiates CARD filament formation. **e** Schematic diagram of UPA oligomerization facilitating NLRP1 CARD filament formation. Domains are color-coded as in **a**.

NLRP1 was the first molecule identified as an innate immune sensor that can assemble an inflammasome.^6^ Humans have only NLRP1, whereas mice have NLRP1a, NLRP1b, and NLRP1c, with human NLRP1 and murine NLRP1b being the most functionally similar: they share related inflammasome functions but differ in domain organization and regulatory mechanisms.^49–51^ Human NLRP1 consists of a pyrin domain (PYD); a NACHT domain with ATPase activity that mediates oligomerization; and a leucine-rich repeat (LRR) domain, followed by a function-to-find domain (FIIND) and a caspase activation and recruitment domain (CARD) (Fig. 1a). In contrast, murine NLRP1b lacks the N-terminal PYD and exhibits a distinct activation mechanism.^49–51^ In humans, under resting conditions, NLRP1 undergoes constitutive autoproteolysis within the FIIND, generating two fragments: an N-terminal PYD-NACHT-LRR-ZU5 fragment that undergoes proteasomal degradation and a C-terminal UPA-CARD fragment^49,50^ (Fig. 1a). The released UPA-CARD fragment is stabilized through interactions with dipeptidyl peptidase 8 (DPP8) or DPP9, preventing inflammasome formation. In the presence of Val-boroPro (VbP), an inhibitor of DPP8/DPP9, this stabilization is disrupted, allowing the release of the UPA-CARD fragment (NLRP1CT) from its autoinhibitory state to promote inflammasome assembly; this assembly involves interaction with the adaptor molecule ASC, which contains both PYD and CARD regions, to connect sensor molecules to caspase-1. As a result of complex assembly, caspase-1 is activated and inflammatory cell death occurs^52,53^ (Fig. 1a). In contrast, murine NLRP1b is activated primarily through pathogen-encoded proteases, such as anthrax lethal factor, which cleave the N-terminus and promote proteasome-dependent degradation to release the C-terminal CARD.^49,51^ However, murine NLRP1b is also sensitive to DPP8/9 inhibition by VbP. Thus, DPP8/9-mediated regulation appears to be conserved across species, even though the primary activation pathways differ.^52,53^

These unique modes of action, exclusive to NLRP1 among all the nucleotide-binding domain and leucine-rich repeat-containing proteins (NLRs), were unclear until the recent breakthroughs provided by cryo-EM structures of human and murine NLRP1 bound to DPP9^54,55^ (Table 2). However, the NACHT, LRR, and CARD domains were poorly resolved, likely due to structural flexibility, with only the FIIND (ZU5 and UPA domains) being clearly visible.^54,55^ Here, we will focus our discussion primarily on the human NLRP1–DPP9 complex.

**Table 2:** Experimental evidence supporting inflammasomes and PANoptosomes.

Inflammasome/PANoptosome	Structural evidence	Biochemical evidence	Genetic evidence	Imaging evidence in single cells	Example references
NLRP1 inflammasome	Sensor structure	In vitro reconstitution and co-IP	Yes	Core component colocalization	^54,62^
NLRP3 inflammasome	Sensor structure and partial inflammasome assembly	In vitro reconstitution and co-IP	Yes	Core component colocalization	^90,91,95–97,307^
NAIP/NLRC4 inflammasome	Sensor structure	In vitro reconstitution and co-IP	Yes	Core component colocalization	^168,297^
AIM2 inflammasome	Sensor structure	In vitro reconstitution and co-IP	Yes	Core component colocalization	^196^
Pyrin inflammasome	Sensor structure	In vitro reconstitution and co-IP	Yes	Core component colocalization	^237^
ZBP1-PANoptosome	Sensor structure (limited to domains)	In vitro reconstitution and co-IP	Yes	Core component colocalization	^25,33,34,299,300^
NLRP3-PANoptosome	Sensor structure	In vitro reconstitution and co-IP	Yes	Core component colocalization	^30,90,91,95–97,307^
AIM2-PANoptosome	Sensor structure	In vitro reconstitution and co-IP	Yes	Core component colocalization	^24,196,197^
RIPK1-PANoptosome	Sensor structure (limited to domains)	In vitro reconstitution and co-IP	Yes	Core component colocalization	^26,302,303^
NLRC5-/NLRP12-PANoptosome	Sensor structure (limited to domains)	In vitro reconstitution and co-IP	Yes	Core component colocalization	^27,28,306^

The cryo-EM structures show that DPP9 plays a critical role in maintaining NLRP1 in its inhibitory state.^54,55^ It directly binds to both full-length NLRP1 (NLRP1FL) and NLRP1CT. In NLRP1FL, the UPA domain adopts β-sheet conformations with ZU5, locking the UPA-CARD in an inactive state, even after FIIND autoproteolysis (Fig. 1b). In contrast, the UPA domain in NLRP1CT has an unstructured N-terminal segment (S1212–N1224) containing a DPP9 substrate motif. This segment threads into the active-site tunnel of DPP9 (Fig. 1c). By binding to the unstructured segment of NLRP1CT, DPP9 prevents UPA-mediated polymerization of the UPA-CARD fragment. This steric hindrance effectively inhibits complex formation and maintains NLRP1 in its inactive state. The binding of DPP9 to the UPA-CARD fragment depends on the simultaneous interaction of DPP9 with NLRP1FL. This means that DPP9 can inhibit activation only when sufficient NLRP1 is present, highlighting its role in maintaining a balance between NLRP1FL and NLRP1CT.^54,55^ Activation by VbP disrupts this balance. VbP competes with the N-terminal segment (S1212–N1224) of NLRP1CT’s UPA domain and displaces it from the DPP9 active site (Fig. 1d). This process releases the UPA-CARD fragment, allowing the CARD to interact with the CARD of the apoptosis-associated speck-like protein containing a CARD (ASC) and form CARD filaments. These filaments ultimately drive complex assembly.^54,56,57^

Recent biochemical and structural studies have identified additional regulatory layers in NLRP1 activation. Oxidized thioredoxin 1 (oxTRX1) directly binds the NACHT-LRR region of human NLRP1.^58,59^ This interaction appears to stabilize the autoinhibited conformation under basal redox conditions, preventing premature NLRP1 activation. Additionally, oxTRX1 binding is proposed to modulate the accessibility of the N-terminus to proteasomal degradation, a process essential for releasing the C-terminal FIIND-CARD fragment. Because N-terminal degradation is a prerequisite for NLRP1 activation, oxTRX1 acts as a redox-responsive checkpoint.^58,59^ In addition to oxTRX1-dependent stabilization, NLRP1 is regulated by post-translational modifications (PTMs) that influence its activation.^60^ NLRP1 undergoes ubiquitin-mediated N-terminal degradation, which is required to release the C-terminal FIIND-CARD fragment.^60^

Mechanistic principles governing NLRP1 activation become clearer when viewed alongside CARD8, a closely related FIIND-containing sensor.^50,57^ Like NLRP1, CARD8 undergoes FIIND autoproteolysis and requires N-terminal degradation to release its C-terminal CARD fragment.^50^ Once liberated, this fragment directly engages caspase-1, enabling inflammasome assembly in response to distinct cellular stresses.^50^ These mechanistic parallels highlight shared principles between NLRP1 and CARD8, including FIIND-dependent regulation, proteasome-mediated N-terminal processing, and CARD-driven signaling.^61,62^

Structural studies of CARD filaments formed following NLRP1 activation have provided valuable insights into the mechanisms of oligomerization among inflammasome components. Although the NLRP1 CARD alone cannot form stable filaments, UPA oligomerization enhances CARD multimerization and facilitates ASC recruitment.^54,57^ Cryo-EM analysis of the NLRP1CT fragment shows well-defined density for the CARD, whereas the UPA domain appears more flexible and loosely arranged around the filament periphery (Fig. 1e).^63^ The UPA-CARD complex forms a two-layered filament structure, with the CARD as the inner core and UPA spiraling around it. This creates a corncob-like appearance with approximately 11–12 subunits per turn and a helical rise of 5.6 nm. The complex measures 14–15 nm in diameter, with an 8-nm UPA spiral ring encircling the CARD core. A linker region (amino acids 1213–1223 in FIIND-UPA and 1466–1473 in CARD) contributes to the structure’s helical symmetry, while hydrophobic residues 1278–1279 facilitate FIIND-UPA self-oligomerization in a “side-by-side” pattern.^63^

These filament structures suggest that the CARD of NLRP1CT recruits the CARD of ASC through charge complementarity at the filament cross-sections. However, the NLRP1FL CARD is predicted to be unable to recruit the CARD of caspase-1 directly due to a lack of structural and charge compatibility at the interface. This highlights the critical role of the UPA subdomain in NLRP1 oligomerization and filament formation, which enhances CARD polymerization and ASC recruitment.^54,57,63^

### Targeting NLRP1 and its role in diseases

The involvement of NLRP1 in diseases has been broadly examined in prior reviews,^64–66^ and we briefly discuss these roles here. NLRP1 has critical roles in neuroinflammation, with studies linking it to a range of neuronal pathologies, including ischemic brain injury, subarachnoid hemorrhage, and traumatic brain injury (TBI). In patients with TBI, NLRP1 inflammasome components have been identified in exosomes isolated from cerebrospinal fluid, suggesting a potential therapeutic application for blocking inflammasome activation.^67^ Mechanistically, NLRP1 activation also leads to cleavage of X-linked inhibitor of apoptosis protein (XIAP) and promotes extracellular acidosis related to cortical neuronal damage.^68,69^

Beyond the central nervous system, polymorphisms in the *NLRP1* gene are associated with autoimmune and autoinflammatory conditions, including vitiligo, Addison’s disease, atopic dermatitis, psoriasis vulgaris, and celiac disease.^70–72^ The M1184V mutation is one of the most extensively studied NLRP1 variants and has been linked to diabetes, inflammatory bowel disease, and asthma.^73^ This mutation stabilizes NLRP1 by maintaining FIIND in a monomeric conformation, thereby decreasing NLRP1 complex formation and inflammasome activation.^73^ Another mutant variant, L155H, significantly increases susceptibility to vitiligo and autoimmune disorders.^73^ Additional mutations in the PYD, such as A54T, A66V, and M77T, have been associated with skin and respiratory conditions such as familial keratosis lichenoides chronica, skin cancer, and recurrent respiratory papillomatosis.^74,75^ In mice, the Q593P mutation in NLRP1a drives inflammation and immune cell infiltration, reinforcing the role of NLRP1 in regulating inflammatory responses.^76^ Therefore, targeting NLRP1 represents a promising therapeutic strategy not only for preventing inflammatory diseases, but also for enhancing cancer immunotherapy.^77,78^

In this context, structure-based drug design is crucial for targeting NLRP1. Advances in cryo-EM and other complementary structural biology techniques have provided valuable insights, opening the possibility of developing small-molecule inhibitors, peptides, and antibodies that can disrupt key interactions necessary for inflammasome assembly and activation.^54–57,63,79^ Targeting key NLRP1 interactions, such as those with DPP8/9 or its polymerization into a complex with ASC, caspase-1, and potentially other molecules, could be an effective strategy to treat NLRP1-dependent diseases. Additionally, disease-associated variants such as M1184V or L155H present unique opportunities for mutation-specific drug design, allowing precision targeting of dysregulated NLRP1 in susceptible individuals. As structural understanding improves, the ability to develop effective, tailored therapies against NLRP1-driven inflammation will increase.

## NLRP3

### Structural features of NLRP3

NLRP3 is one of the most extensively studied cytosolic innate immune sensors (Table 2); however, despite intensive investigations, its activation mechanism remains enigmatic. NLRP3 can be activated by a broad spectrum of stimuli — including physiologically relevant infections, PAMPs, and DAMPs — which suggests an indirect mode of activation reliant on sensing cellular perturbations rather than specific ligand binding.^80,81^ Recent studies have demonstrated that, in addition to its well-known role as an inflammasome sensor, NLRP3 also drives PANoptosome formation and acts as an integral component of multiple PANoptosomes.^25–28,30^ Moreover, NLRP3 can be activated by the non-canonical inflammasome pathway, which will be discussed in the next section.

**Fig. 2:**
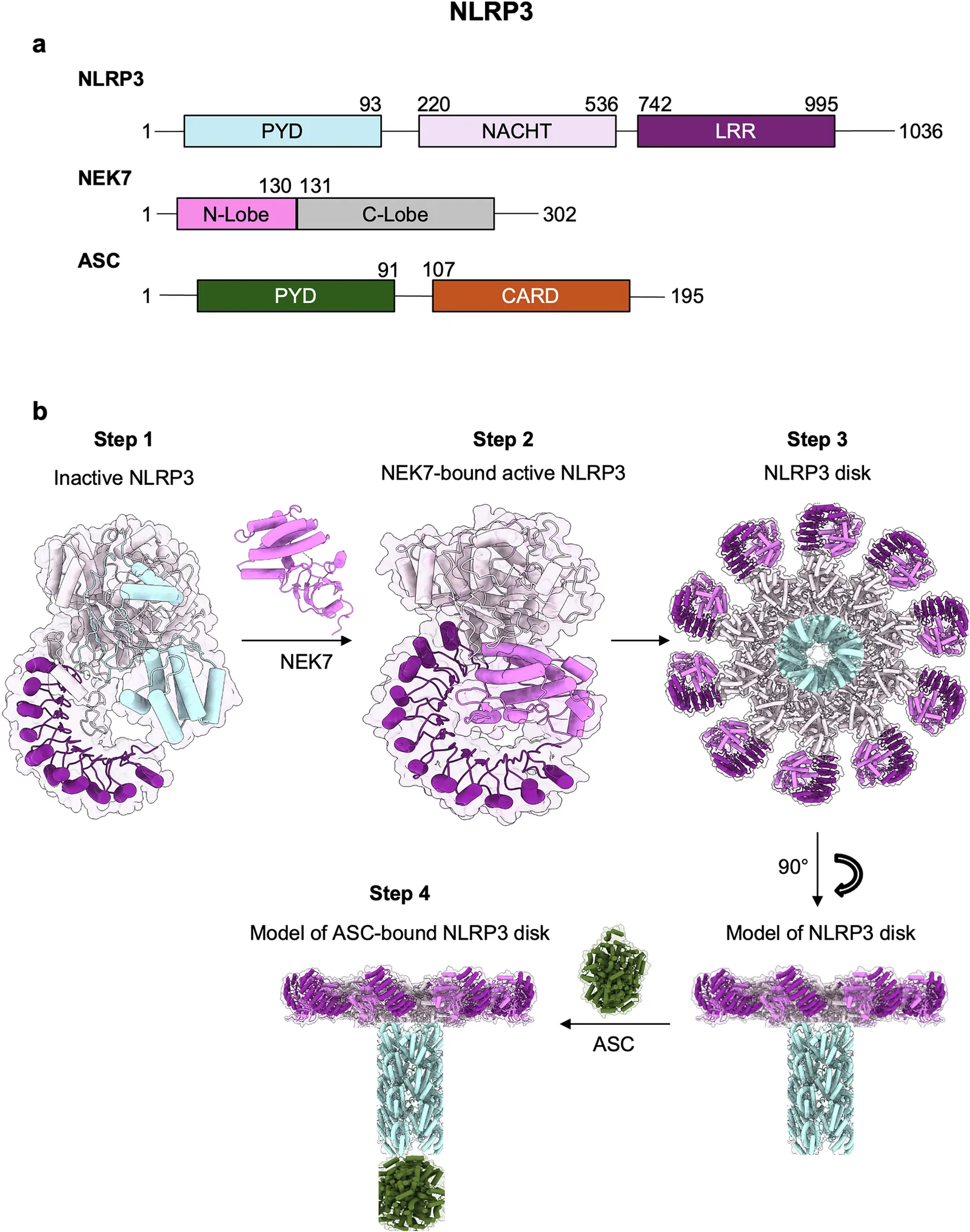
Structural insights into NLRP3 activation. **a** Domain architecture of NEK7, NLRP3, and ASC. Residue numbers for domain boundaries are indicated based on the human protein sequences. **b** The proposed model of NLRP3 activation. Step 1: in the cytosol, the NLRP3 protein is present in an inactive or cage-like form (PDB: 7PZC). Step 2: conformational change in NLRP3 upon binding to NEK7 (PDB: 6NPY). Step 3: the activated NLRP3 rearranges into a disk (PDB: 8EJ4). Step 4: the adaptor protein ASC (PDB: 2KN6) is recruited to the NLRP3 disk via homotypic PYD–PYD interactions, resulting in a PYD–PYD filament that oligomerizes the CARDs of ASC molecules to mediate caspase-1 recruitment. Domains are color-coded as in **a**.

Structurally, NLRP3 consists of three main domains: a PYD at the N-terminus, which recruits proteins for complex formation; a central NACHT domain; and an LRR domain at the C-terminus^82^ (Fig. 2a). NLR proteins are challenging to purify and stabilize under in vitro conditions due to their intrinsic tendency to form higher-order oligomers. This often leads to aggregation, creating a bottleneck for determining their structures at atomic resolution. To overcome these challenges, the structural elucidation of NLRP3 has been facilitated by the use of stabilizing agents, such as ADP, the inhibitor MCC950, and the NIMA-related kinase 7 (NEK7).^83–90^

Before the advent of cryo-EM, the structure of NLRP3 PYD was determined using both X-ray crystallography and solution-state nuclear magnetic resonance (NMR).^91,92^ Both techniques indicated a similar overall architecture of the PYD with six helices (α1–α6) and five connecting loops. More recently, the cryo-EM structures of NLRP3 in inactive and NEK7-bound states have been determined, providing key insights into its regulation^83–85,87^ (Fig. 2b). In the inactive ADP-bound state, NLRP3 adopts an “earring”-shaped conformation, similar to NLRC4 and NAIP5.^87,93^ These findings support a model where NEK7 binds after NLRP3 undergoes conformational rearrangement. NEK7 specifically interacts with the NLRP3 nucleotide-binding domain (NBD) and LRR domain via its C-terminal lobe, while the N-terminal lobe remains disordered. Mutagenesis studies confirm that the LRR is critical for NEK7 binding, with minimal contribution from the NBD.^87^ NEK7 binding is enhanced by ADP and MCC950, suggesting that ligands and cofactors stabilize NLRP3 for structural analysis.^87^ During activation, the NBD rotates away from the NEK7 binding site, allowing NEK7 to bind^90^ (Fig. 2b). However, recent studies have shown that NEK7 may not be strictly required for NLRP3 activation in human macrophages,^94^ suggesting that further investigation may be required to fully understand the NLRP3 structure–function relationship.

Structural studies have also demonstrated that NLRP3 assembles into cage-like oligomers, with human NLRP3 forming decamers and mouse NLRP3 forming oligomers ranging from dodecamers to hexadecamers.^88,89,95^ The formation of cages, which shield the PYDs and prevent premature activation, is driven by interactions among LRR domains and PYD–PYD contacts.^88,89,95^ Upon activation, NLRP3 complexes have been visualized as disk-shaped oligomers with NACHT domains at the center and LRR domains bound to NEK7 forming the outer ring^90^ (Fig. 2b). PYD filaments extend outward, engaging ASC via PYD–PYD interactions to initiate downstream signaling (Fig. 2b). Thus, activation of NLRP3 involves conformational shifts between inactive (ADP-bound) and active (ATP-bound) states, with NEK7 unlocking the cages for assembly.^90^ Complementing these findings, cryo-ET studies demonstrated that NLRP3 activation promotes the formation of dense, branched ASC filament networks.^96^ Another study further showed that activated NLRP3 accumulates in condensates at and around the microtubule-organizing center before cell death.^97^ Together, these observations suggest that complex assembly involves dynamic spatial reorganization. Small vesicles traffic NLRP3 from the *trans*-Golgi network (TGN),^96–98^ after which NEK7 binding opens NLRP3 cages for active complex assembly.^87^ ASC is then recruited through PYD–PYD interactions, forming aggregates that activate caspase-1 and drive inflammatory signaling.

In conjunction with subcellular localization, NLRP3 is also regulated by PTMs. It undergoes S-palmitoylation at Cys126 by zinc finger DHHC palmitoyltransferase 7 (ZDHHC7), which modulates its trafficking and activation.^99,100^ The S-palmitoylation promotes NLRP3 localization at the TGN, modulates its mobility, and influences higher-order oligomerization. Mutation of ZDHHC7 or the palmitoylation site diminishes NLRP3 activation in macrophages and in vivo models of inflammatory challenge.^100^ Palmitoylation at Cys126 or additional cysteines can promote interactions with NEK7, stabilize oligomeric intermediates, or drive autophagic degradation.^100,101^ Furthermore, NLRP3 is regulated by ubiquitination, phosphorylation, and acetylation.^102^ For example, HDAC10-mediated switching between acetylation and ubiquitination at lysine residues can enhance proteasomal degradation of NLRP3.^102^ In parallel, liquid-liquid phase separation (LLPS) has emerged as a mechanism for NLRP3 activation. Activation signals such as K^+^ efflux drive NLRP3 into liquid-like condensates, initiating inflammasome assembly.^98^ Collectively, these findings suggest that there is a dynamic interplay between organelle trafficking and NLRP3 activation in response to some stimuli. A current hypothesis is that resting NLRP3 resides at the TGN and associates with small vesicles, where it is maintained in a monomeric or chaperone-bound state.^99,102^ Upon stimulation, NLRP3 translocates to dispersed TGN compartments and undergoes higher-order oligomerization, a process suggested to be facilitated by palmitoylation^99^ and to involve phase separation.^98^ This event promotes the recruitment of ASC and caspase-1. While similar trafficking mechanisms have not yet been directly demonstrated for other sensors, it is plausible that vesicle-mediated transport could contribute in other contexts.

In addition to interacting with NEK7 and ASC, co-immunoprecipitation and microscopy studies have shown that NLRP3 also associates with other components of PANoptosomes. Specifically, in the NLRP3-PANoptosome, NLRP3 colocalization with ASC, caspase-8, and RIPK3 has been seen by microscopy.^30^ In the ZBP1-PANoptosome, NLRP3 can be pulled down by co-immunoprecipitation with ASC, caspase-1, caspase-8, caspase-6, RIPK1, and RIPK3;^25,29,43^ in the RIPK1-PANoptosome, it co-immunoprecipitates with ASC, caspase-8, FADD, RIPK3, and RIPK1;^26^ and in the NLRC5- and NLRP12-PANoptosome, NLRP3 can be pulled down with NLRC5, NLRP12, ASC, caspase-8, and RIPK3.^27,28^ Furthermore, genetic and molecular studies show that NLRP3 is the essential sensor required for NLRP3-PANoptosome formation in response to PAMPs and DAMPs; when NLRP3 is genetically deleted, the complex does not form, and the cells are protected from PANoptosis.^30^

While the structural organization of NLRP3 at the sensor level has been extensively defined, the architectures of the full NLRP3 inflammasome and PANoptosome complexes have not yet been resolved and are inferred from genetic, biochemical, and imaging studies. Future structural investigations will shed new light on these interactions. The current structural studies focused on NLRP3 have provided critical insights into the molecular mechanisms of its activation, suggesting central roles for intracellular trafficking and protein–protein interactions in regulating the formation of the active inflammasome complex to drive caspase-1 activation and inflammatory cell death. Future work will be needed to increase our structural understanding of the NLRP3 inflammasome and its role in PANoptosome formation. Additionally, current cryo-EM and cryo-ET studies have been performed with ligand-based triggers that often do not mimic the physiological conditions driving NLRP3 activation in vivo. More clinically relevant triggers will be useful in future studies to define the steps of NLRP3 activation in infectious and inflammatory conditions and to identify therapeutic targets for NLRP3-related diseases.

### Non-canonical inflammasome regulatory pathway

NLRP3 is also activated by the non-canonical inflammasome pathway, which is mediated by caspase-11 in mice and caspase-4/5 in humans.^21,22^ These caspases are expressed as inactive monomers and require dimerization for protease activity.^103,104^ Cytosolic lipopolysaccharide (LPS) directly engages these inflammatory caspases and promotes their oligomerization and activation.^21,22,103,105,106^ Recent work also suggests that activation is influenced by the membrane environment in which LPS is embedded, as lipid packing and membrane curvature can enhance caspase clustering and activation.^107^

Activated inflammatory caspases cleave GSDMD to form membrane pores.^21,22^ This pore formation causes homeostatic disruptions that promote NLRP3 activation.^21,22^ Therefore, non-canonical inflammasome signaling is often viewed as a two-step process: (1) caspase-4/5/11 activation by cytosolic LPS; (2) downstream NLRP3 inflammasome assembly triggered by GSDMD-mediated membrane damage.^21,22^ Whether this can also lead to PANoptosome formation is currently unknown.

Structural studies have provided insights into key steps in this pathway.^108,109^ Crystal structures of inflammatory caspase CARDs show canonical death-domain folds capable of mediating homotypic interactions.^110,111^ These structures support a model in which LPS binding stabilizes CARD-mediated interactions and promotes caspase oligomerization.^79,112^ High-resolution cryo-EM structures have shown caspase-4 bound to cytokine substrates, demonstrating a two-site interaction mechanism. In this model, an exosite located outside the catalytic pocket stabilizes substrate binding.^113,114^ This interaction aligns the substrate correctly within the active site and enables efficient proteolysis.^113,114^ These findings inform how inflammatory caspases engage and process substrates once activated.

Recently, cryo-ET studies provided an in situ view of non-canonical inflammasome signaling within intact cells.^115^ These studies showed the spatial organization of caspase-4 and its association with cellular membranes and organelles.^115^ However, due to resolution limits, cryo-ET does not resolve enzyme–substrate interfaces or ligand-binding modes. Consistent with this limitation, high-resolution structures of full-length, activated caspase complexes are not yet available.

### Targeting NLRP3 and its role in diseases

The role of NLRP3 in human disease has been extensively reviewed previously,^116–119^ and here we focus on select examples that illustrate its diverse involvement across inflammatory, infectious, and metabolic disorders.

NLRP3 is implicated in a wide spectrum of diseases, including infectious diseases, cancer, and monogenic autoinflammatory syndromes such as cryopyrin-associated periodic syndromes (CAPS), as well as more complex conditions like gout, Alzheimer’s disease, Parkinson’s disease, and type 2 diabetes.^9,116–124^ Other studies have shown that NLRP3 may also respond to metabolic stress, such as increased extracellular glucose; environmental irritants, such as silica, asbestos, and ultraviolet B irradiation; and skin irritants, including trinitrophenylchloride, trinitrochlorobenzene, and dinitrofluorobenzene.^125–128^

Due to the variety of triggers that activate it, NLRP3 is central to the pathophysiology of diverse diseases.^124^ In obesity and diabetes, ceramide accumulation in macrophages and adipose tissue drives lipotoxic stress and inflammasome activation, as demonstrated in preclinical models.^138^ NLRP3 is also linked to conditions such as irritable bowel syndrome, colitis, and nonalcoholic fatty liver disease, in which cholesterol crystals act as a trigger.^139,140^ NLRP3 is further implicated in a broad spectrum of infections; for example, *Mycoplasma pneumoniae* triggers ADP-ribosylation-dependent NLRP3 activation in preclinical studies.^141^ In SARS-CoV-2 infection, NLRP3 activation has been linked to increased disease severity based on clinical observations.^142^ Mutations in NLRP3 are also responsible for CAPS, a group of rare, inherited inflammatory disorders. CAPS encompasses a spectrum of diseases, including familial cold autoinflammatory syndrome, Muckle-Wells syndrome, and the most severe form, neonatal-onset multisystem inflammatory disease (NOMID), also referred to as chronic infantile neurological, cutaneous, and articular (CINCA) syndrome.^122,123,143^

The role of NLRP3 in cancer is complex and context-dependent, with evidence supporting both tumor-promoting and tumor-suppressive functions.^124^ For example, in preclinical models of gastric cancer, NLRP3 enhances cell differentiation through JNK signaling.^129^ Clinical and preclinical evidence in colorectal cancers shows that NLRP3 exerts a protective effect by promoting IL-18-dependent natural killer (NK) cell-mediated tumor surveillance.^130^ Elevated NLRP3 expression correlates with poor prognosis across multiple cancers, including head and neck, lung, bladder, and pancreatic carcinomas.^129,131–133^ In glioblastoma, inhibition of NLRP3 improves survival outcomes during radiation therapy in animal models.^134^ Furthermore, polymorphisms in NLRP3 and related molecules (IL-1β, IL-18, and CARD8) have been linked to leukemias and reduced survival rates in clinical studies.^135,136,137^ Polymorphisms in NLRP3 have also been identified in sporadic cases of malignant melanoma, including nodular subtypes.

Based on this understanding of the molecular basis of NLRP3-driven diseases, there is growing interest in developing targeted therapeutics that are effective across physiological and pathological contexts. Structural biology has begun to elucidate key elements of NLRP3 architecture and activation, which aids in therapeutic development. For example, cryo-EM and cryo-ET studies have defined critical interaction surfaces and conformational transitions that can now be targeted with small molecules, peptides, or biologics.^87–89,95–97,144^

One notable example is MCC950, a small-molecule inhibitor that has been widely studied for its ability to block NLRP3 activation.^86^ MCC950 stabilizes NLRP3 in its inactive conformation by binding to the NACHT domain, preventing ATP hydrolysis and subsequent oligomerization.^145^ Structural studies have established MCC950 as a valuable tool for dissecting NLRP3 activation mechanisms and identifying possible target sites for drug design.^87–89^ However, clinical development of MCC950 has faced limitations due to potential off-target toxicity and immune suppression, as well as its inability to inhibit certain CAPS-driving NLRP3 variants.^146^ Given the high level of interest in therapeutically targeting NLRP3, other NLRP3 inhibitors, such as CY-09 and OLT1177 (dapansutrile), have been developed, with distinct mechanisms of action that include blocking ATP binding or reducing inflammasome assembly.^147–150^ Additionally, NEK7 degraders represent an alternative upstream strategy, suppressing NLRP3 activation by disrupting the essential NLRP3–NEK7 interaction.^151–153^ Together, these approaches illustrate both direct and regulatory strategies to modulate NLRP3, highlighting translational opportunities for therapeutic intervention in inflammatory diseases. Consistent with this, several NLRP3-targeted agents have shown preclinical efficacy^154^ and advanced to clinical evaluation for autoinflammatory and metabolic diseases.^86,145,148,149^ Additional details of these NLRP3 inhibitors have been extensively reviewed elsewhere.^155^

In addition, understanding how disease-associated mutations alter NLRP3 structure and function can guide personalized therapeutic strategies, especially for patients with CAPS or other conditions in which NLRP3 is mutated. Integrating structural data with biochemical and cellular assays will accelerate the discovery of potent, selective NLRP3 modulators that could transform the design of next-generation inhibitors for the treatment of chronic inflammatory, metabolic, infectious, and neoplastic disorders.

## NAIP**–**NLRC4

### Structural features of NAIP–NLRC4

**Fig. 3:**
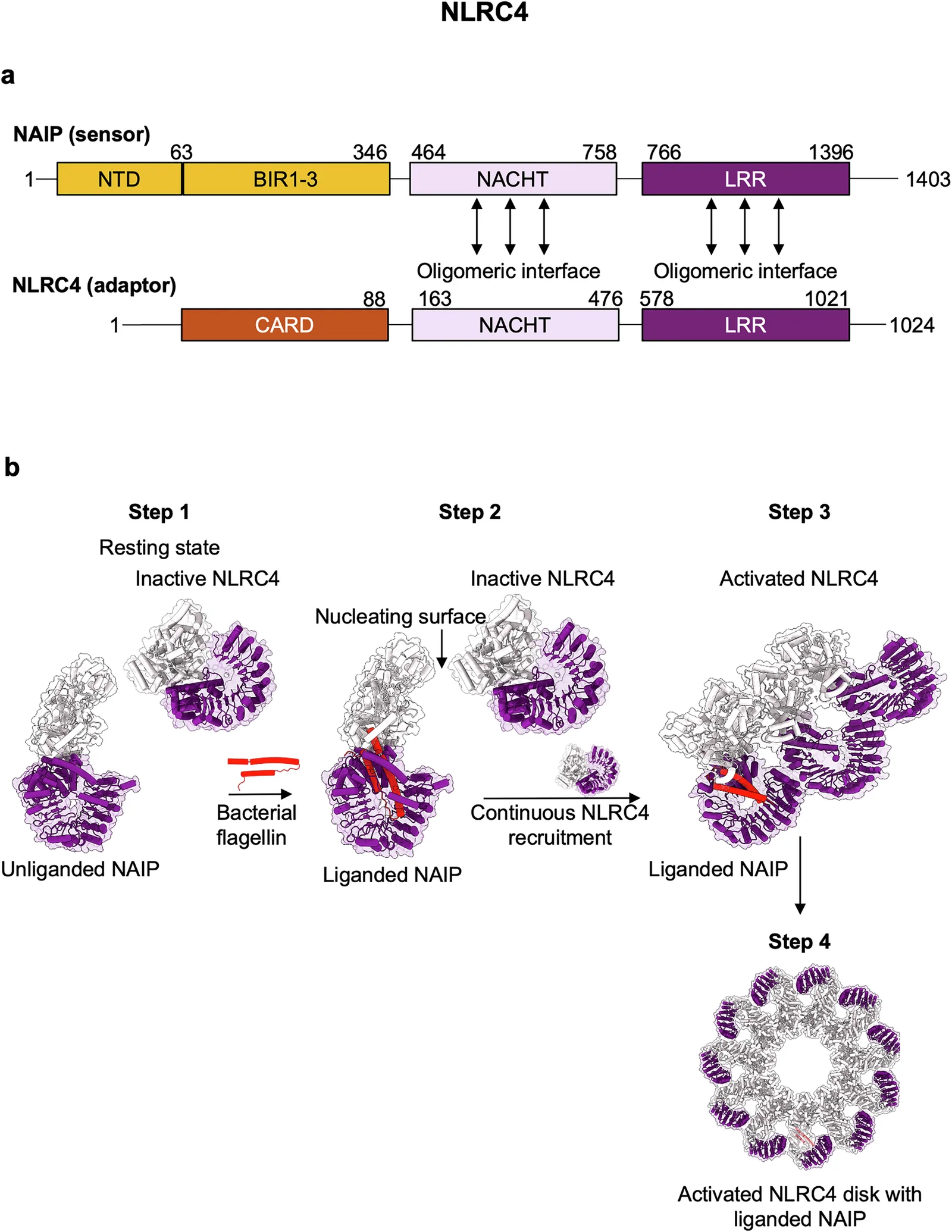
Structural insights into NAIP/NLRC4 activation. **a** Domain architecture of NAIP and NLRC4. Oligomeric interfaces are indicated by black arrows. Residue numbers for domain boundaries are indicated based on the mouse protein sequences. **b** The proposed model of NAIP/NLRC4 assembly. Step 1: the unliganded NAIP (NAIP5 is shown as an example; PDB: 7RAV) and NLRC4 (PDB: 4KXF) are both in inactive/resting states. Step 2: the interaction with bacterial flagellin induces the activation of NAIP5 (PDB: 5YUD). The nucleating surface exposed by these conformational changes is shown. Step 3: the activation of NLRC4 causes self-propagation and leads to continuous recruitment of NLRC4 (PDB: 6B5B). Step 4: the activated NAIP/NLRC4 disk. Domains are color-coded as shown in **a**.

NLR family apoptosis inhibitory proteins (NAIPs) are cytosolic receptors that detect specific bacterial protein ligands and recruit NLRC4 to form NAIP–NLRC4 inflammasomes and cell death complexes.^156–160^ Humans express a single NAIP, while mice express six. In mice, NAIP1–6 have distinct specificities. For example, NAIP5 and NAIP6 recognize bacterial flagellin, while NAIP1 and NAIP2 detect needle and inner rod proteins of bacteria, respectively.^156^ In contrast, the single *NAIP* gene in humans produces a protein capable of recognizing both flagellin and needle-like proteins.^156,161,162^ Unlike other NLRs, NAIPs contain three baculovirus inhibitor-of-apoptosis repeats (BIRs) in their N-terminal regions^156,161^ (Fig. 3a). Upon ligand recognition, NAIPs undergo conformational changes that promote their interaction with NLRC4, triggering assembly of the complex.^163,164^ NLRC4 is composed of three primary domains: the N-terminal CARD, the central NACHT domain, and the C-terminal LRR domain (Fig. 3a).

Structural and biochemical studies have shed light on the assembly and activation mechanisms of NAIP–NLRC4^165,166^ (Table 2). The first reported crystal structure of inactive NLRC4 identified a closed conformation stabilized by extensive intramolecular interactions, marking the first high-resolution structure of an NLR protein.^93^ NLRC4 activation is initiated when a bacterial ligand, such as the inner rod protein PrgJ from *Salmonella enterica* serovar Typhimurium, binds to the corresponding NAIP receptor.^167^ Cryo-EM studies of the PrgJ–NAIP2–NLRC4 complex provided valuable insights into inflammasome assembly. In these studies, an NLRC4 construct lacking the CARD forms a disk-shaped oligomeric complex containing 10–12 subunits, with a single copy of ligand-bound NAIP per disk. These findings support a nucleation-driven mechanism, in which ligand-bound NAIP undergoes a conformational rearrangement that exposes a nucleating interface to recruit and activate adjacent NLRC4 monomers^168,169^ (Fig. 3b). This activation involves structural rearrangement within the NACHT domain of NLRC4, shifting it from a closed, autoinhibited conformation to an open state. This transition drives the stepwise assembly of the disk-shaped inflammasome, leading to caspase-1 recruitment via CARD–CARD interactions^168,169^ (Fig. 3b).

Additional cryo-EM structures of flagellin–NAIP5–NLRC4 complexes further validated this model.^170–172^ For instance, one study reported the structure of NAIP5 bound to full-length *Legionella* flagellin, with two NLRC4 subunits, at 5.2 Å resolution.^172^ Similarly, the structure of NAIP5 in complex with a truncated *Salmonella* flagellin and an oligomerization-deficient NLRC4 mutant has been reported at 4.3 Å resolution.^171^ In both structures, the C-terminal α-helix of flagellin inserts into a deep pocket formed by multiple NAIP5 domains, triggering NAIP5 activation and promoting NLRC4 recruitment. Taken together, these structural insights suggest that NLRC4 remains in an autoinhibited, closed conformation when inactive; upon activation, it oligomerizes to form a complex with ASC to drive inflammation and cell death (Fig. 3b).

Compared with NLRP3, NLRC4 appears to rely less on post-translational regulation. Phosphorylation at Ser533 was initially proposed as a priming event that enhances responsiveness to NAIP engagement.^173^ However, later genetic studies showed that this modification is not strictly required for inflammasome activation and may instead modulate signaling.^174^ Ubiquitination has also been implicated in regulating NLRC4, although its effects remain unclear.^175,176^ Together, these observations suggest that NAIPs are the primary drivers of NLRC4 activation, with PTMs acting as secondary modulators.

### Targeting NLRC4 and its role in diseases

The functions of NAIP–NLRC4 activation in host defense and disease have been thoroughly discussed in previous reviews,^177–180^ and we briefly discuss these roles here. The NLRC4 inflammasome plays a well-established role in innate immune responses to bacterial infection and has also been implicated in inflammatory disorders and cancer. To evade detection, several bacterial pathogens have evolved strategies that inhibit NAIP–NLRC4 activation. For example, *Salmonella* Typhimurium employs two distinct *Salmonella *pathogenicity islands (SPIs): SPI-1, which facilitates invasion of intestinal epithelial cells and delivers type III secretion system components that are recognized by NAIPs; and SPI-2, which enables intracellular survival and replication in macrophages.^159,181^ *Pseudomonas aeruginosa* expresses the effector molecules ExoU, ExoY, ExoT, and ExoS to enhance its infectivity in host cells. Among them, ExoU has been shown to trigger caspase-1-independent cell death, potentially interfering with NAIP–NLRC4 inflammasome assembly.^182^ *Listeria monocytogenes* also prevents NLRC4 activation by downregulating flagellin via the transcriptional regulator MogR.^183^

Beyond infections, NLRC4 has been linked to cancer biology, where it exhibits both tumor-promoting and tumor-suppressive roles.^184–187^ On the one hand, NLRC4 activation has been implicated in promoting vascular endothelial growth factor-A (VEGF-A) production, which contributes to angiogenesis and cancer progression, as well as to intracerebral hemorrhage.^188^ On the other hand, NAIP–NLRC4 activation has tumor-suppressive effects in mouse models of colitis-associated colorectal cancer, making the NAIP–NLRC4 pathway a potential therapeutic target for cancer treatment.^184^ Its dual roles in promoting angiogenesis in certain cancer types and serving as a tumor suppressor in colorectal cancer highlight the complex contributions of NLRC4 across different disease contexts.^185–187^

Genetic studies have also identified gain-of-function mutations in NLRC4 that result in spontaneous inflammasome activation and autoinflammatory syndromes. Mutations such as T337A, V341A, H443P, and F171A result in gain-of-function effects, triggering spontaneous inflammasome activation.^189^ The H443P mutation is linked to familial autoinflammatory diseases and promotes caspase-8-dependent cell death.^190,191^ The F171A mutation disrupts interactions with residue T337, promoting inflammasome activation and leading to NLRC4-induced enterocolitis.^192^ Additionally, a T337A mutation in the HD1 domain has been associated with macrophage activation syndrome and elevated IL-18 levels.^193^ Another somatic mutation, T177A, was identified in a patient with NOMID-like symptoms, expanding the clinical spectrum of NLRC4-associated diseases.^194^

Given its importance in diseases, NLRC4 stands out as a promising therapeutic target. Improved structural understanding of its activation mechanisms, particularly the transition from an autoinhibited to an active oligomeric state upon engagement by ligand-bound NAIPs,^93,168–172^ provides a strong foundation for structure-based drug design aimed at modulating NLRC4 activity. Therapeutic strategies could focus on stabilizing the autoinhibited conformation of NLRC4 or interfering with its nucleation and oligomerization interfaces. For instance, small molecules or peptides could be engineered to mimic NAIP-binding motifs or selectively block oligomerization sites, thereby preventing inflammasome assembly. Additionally, structure-guided approaches could enable the development of mutation-specific inhibitors targeting hyperactive variants such as T337A, H443P, or F171A. These strategies hold potential for precision therapies aimed at treating NLRC4-driven inflammatory diseases and inflammation-associated cancers.

## AIM2

### Structural features of AIM2

**Fig. 4:**
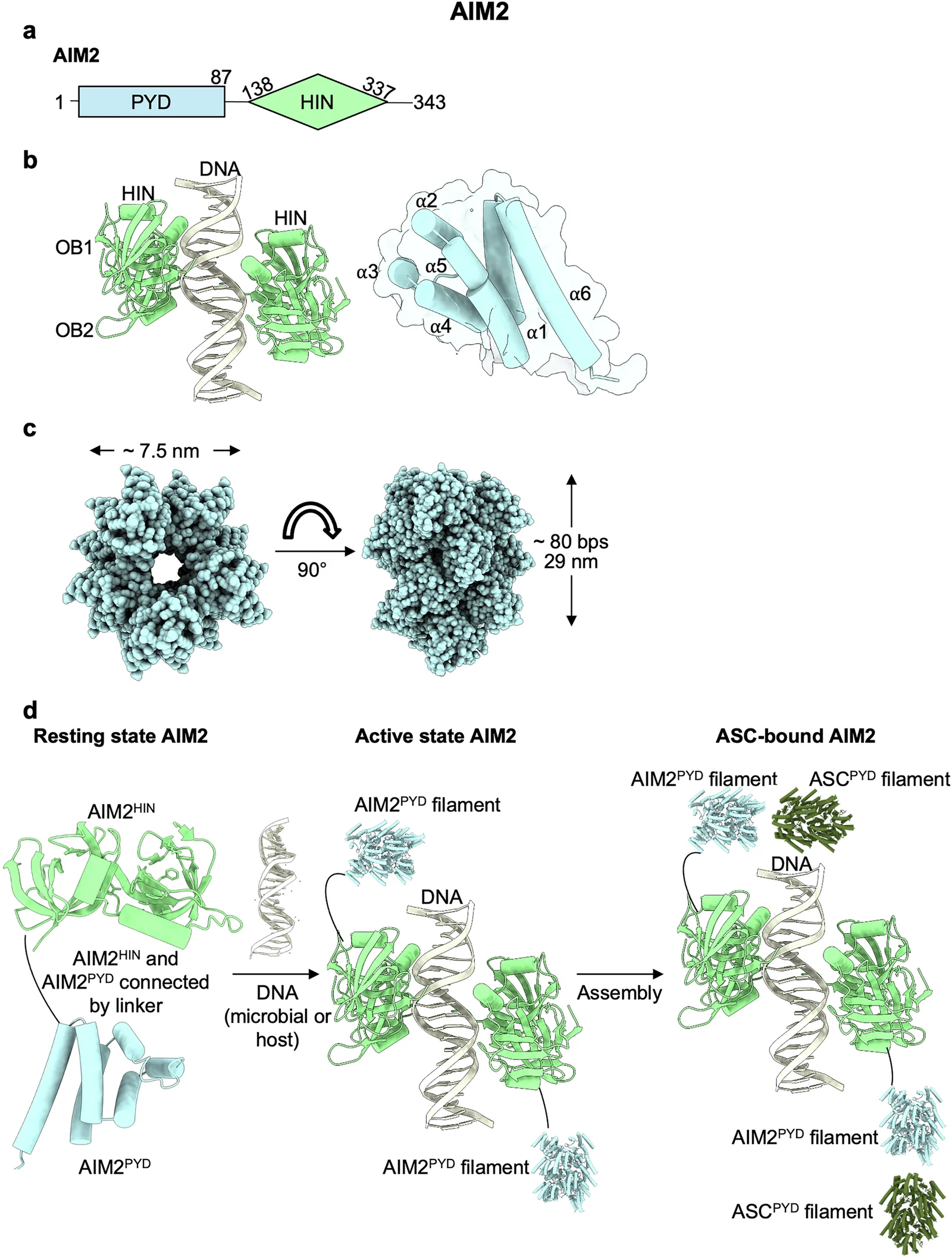
Structural insights into AIM2 activation. **a** Domain architecture of AIM2. Residue numbers for domain boundaries are indicated based on the human protein sequence. **b** Left, crystal structure of the AIM2^HIN^ domain in complex with dsDNA showing the two tandem OB domains connected by a linker (PDB: 3RN2). Right, crystal structure of AIM2^PYD^ showing the six-helix bundle conformation, a characteristic feature of DD superfamily members (PDB: 3VD8). **c** Cryo-EM structure of AIM2^PYD^ filaments. Top view (left panel) and side view (right panel) of the AIM2^PYD^ filaments. DNA with a length of ~80 bp is optimal for AIM2 activation. To accommodate this DNA length, the AIM2^PYD^ filaments have a diameter of ~7.5 nm and a length of ~29 nm (PDB: 6MB2). **d** Schematic model of AIM2^PYD^ filaments forming the structural scaffolds for the oligomerization of ASC^PYD^. Upon sensing dsDNA, AIM2 binds to the DNA via its HIN domain, which wraps around the dsDNA, releasing AIM2 from autoinhibition. This enables AIM2^PYD^ to assemble into helical filaments along the DNA, serving as a scaffold for ASC^PYD^ oligomerization and AIM2 complex formation. Domains are color-coded as in **a**. Solid black lines indicate the flexible linker region between the PYD and HIN domains.

AIM2 is a cytosolic sensor that detects double-stranded DNA (dsDNA) from microbial or host sources.^195^ AIM2 belongs to the PYD and interferon-inducible (HIN) domain-containing (PYHIN) family and is characterized by the presence of the PYD at the N-terminus and the hematopoietic, HIN domain at the C-terminus, connected by a long linker^196,197^ (Fig. 4a). Binding of dsDNA to the HIN domain releases AIM2 from autoinhibition, allowing it to oligomerize and assemble higher-order signaling complexes, such as inflammasomes or PANoptosomes^24,198^ (Table 2). The PYD of AIM2 then recruits the adaptor protein ASC via homotypic PYD–PYD interactions, leading to ASC filament formation. In the context of inflammasome formation, the CARD of ASC subsequently engages and activates pro-caspase-1, initiating the maturation of IL-1β and IL-18 and driving cell death.^199^ Genetic, molecular, and biochemical data also show that AIM2 forms the AIM2-PANoptosome to drive PANoptosis,^24^ and future structural studies will improve our understanding of this complex.

Structural studies, including the elucidation of the crystal structures of AIM2^HIN^ ^197^ and AIM2^PYD^,^196^ as well as the cryo-EM structure of AIM2^PYD^ filaments,^200^ have provided crucial mechanistic insights into AIM2 and its complex assembly, particularly for inflammasome formation. The AIM2 HIN domain (~200 amino acids) contains two tandem oligonucleotide/oligosaccharide-binding (OB) folds connected by a flexible linker composed of two short α-helices or an extended loop. Canonical OB folds consist of five β-strands that assemble into two sheets, forming a stable interface through hydrophobic interactions. The DNA-binding surface of the HIN domain is positively charged, enabling electrostatic interaction with the sugar-phosphate backbone of dsDNA^197^ (left panel, Fig. 4b). Furthermore, the N-terminus of the HIN domain is positioned away from the DNA-binding site, possibly to facilitate the interaction of the N-terminal PYD of AIM2 with the adaptor protein ASC.^197^ Crystallographic analysis of the AIM2^HIN^ domain bound to vaccinia virus DNA demonstrated that ~80 base pairs (bp) of dsDNA are required for efficient inflammasome activation and IL-1β production. Each HIN domain occupies 4 bp of dsDNA; thus, ~20 AIM2^HIN^ domains wrap around the 80 bp of dsDNA with an axis tilt of 35°. Collectively, these results suggest a framework in which the intact DNA-binding interface is required for effective AIM2 activation^197^ (left panel, Fig. 4b).

The crystal structure of AIM2^PYD^ shows a six-helix bundle conformation, a typical feature of death-domain (DD) superfamily members^196^ (right panel, Fig. 4b). AIM2^PYD^ shares structural similarity with other DD superfamily members, particularly the PYDs of ASC and NLRP3, with root-mean-square deviation (RMSD) values of 1.8 Å and 1.6 Å, respectively.^196^ AIM2^PYD^ features a short, flexible α3 helix and an extended α6 helix, with the α3 helix stabilized by residue K26 through hydrogen bonding.^196,197^ This lysine (K26) is conserved among AIM2 proteins across different species, supporting its structural and functional importance. The AIM2^PYD^ surface is highly charged, with residues such as K64 and R85 conserved across PYHIN family members. AIM2-specific residues E20 and E21, along with hydrophobic residues F27 and F28, contribute to a unique electrostatic and hydrophobic surface, facilitating filament formation through homotypic PYD–PYD interactions.^196^

Structural and biochemical evidence shows that the AIM2^PYD^ alone is sufficient to self-assemble into filaments in vitro and drive inflammasome activation in cells.^201^ In contrast, the isolated AIM2^HIN^ domain lacks the ability to form ordered macromolecular structures, reinforcing the central role of the PYD in supramolecular complex formation.^202^ AIM2^PYD^ filament formation is highly sensitive to environmental factors, such as pH and ionic strength. At low pH and an ionic strength of < 100 mM, AIM2^PYD^ remains monomeric due to repulsion between its positively charged surfaces and the disruption of hydrophobic interactions.^203^ A similar dual dependence on electrostatic and hydrophobic interactions is observed in other PYDs, including those from ASC and NLRP3.^202,204,205^

Cryo-EM analysis of GFP-tagged AIM2^PYD^ identified filaments with helical symmetry and lengths ranging from ~200 nm to 1 µm, with outer and inner diameters of 90 Å and 20 Å, respectively (Fig. 4c).^200^ A detailed structural analysis suggests that AIM2^PYD^ filament formation is stabilized by three canonical interactions that are found among members of the DD superfamily: type I, type II, and type III. Type I interactions, which primarily drive AIM2^PYD^ filament assembly, involve hydrophobic contacts between helices α1 and α4 of one protomer and helices α2 and α3 of another; type II interactions occur between helix α4 of one protomer and helices α5 and α6 of another protomer; type III interactions are formed between helix α3 of one protomer and helices α1 and α6 of another protomer. Likewise, mutations disrupting type I interactions, such as F27G/F27L or L10A/L11A, can prevent filament formation and favor the monomeric state.^200^

Collectively, the crystal structures of AIM2^HIN ^^197^ and AIM2^PYD^ ^196^ and EM analysis of AIM2^PYD^ filaments^200^ provide a conceptual model for dsDNA-induced AIM2 activation (Fig. 4d). In brief, upon sensing dsDNA of microbial or host origin, AIM2 binds to the DNA via its HIN domain, which wraps around the DNA. This step unlocks AIM2 from its autoinhibited state and allows AIM2^PYD^ domains to come into closer proximity. AIM2^PYD^ then utilizes the linker to assemble into a helical filament alongside the dsDNA. These AIM2^PYD^ filaments serve as the structural scaffold for the oligomerization of the PYDs of ASC, thereby resulting in the assembly of the inflammasome^201^ (Fig. 4d).

Although AIM2 activation is primarily governed by dsDNA binding and PYD filament formation, additional regulatory mechanisms constrain its activity. One study found that TRIM11‑mediated ubiquitination targets AIM2 for proteasomal degradation and acts as a negative regulatory checkpoint.^206^ In addition, interferon-inducible protein 16 (IFI16) family proteins, such as p202, antagonize AIM2 by inhibiting DNA binding or competing for PYD‑filament formation.^207,208^

Overall, while many aspects of AIM2 biology are relatively well defined, key questions remain. It is still unclear how AIM2^PYD^ assembles along dsDNA to initiate filament formation. In addition, although the AIM2 HIN and PYD domains have been resolved at high resolution, the higher-order organization of AIM2-containing PANoptosomes remains structurally undefined. Addressing these gaps will be critical for understanding how AIM2 balances robust inflammatory cell death with tight regulatory control.

### Targeting AIM2 and its role in diseases

The roles of AIM2 in infections, inflammation, and cancer have been extensively reviewed in the literature,^209–211^ and we briefly discuss these roles here. AIM2 is a key component of the innate immune response to pathogens, including bacteria and DNA viruses, but many pathogens have evolved strategies to suppress AIM2 activation. For example, herpes simplex virus 1 (HSV-1) expresses the VP22 protein, which binds DNA to block AIM2 activation.^212^ Similarly, human cytomegalovirus (HCMV) encodes the pUL83 protein to block AIM2 function.^213^ Poxviruses produce a viral PYD-only protein (vPOP) that interferes with AIM2–ASC interactions.^214^ In hepatitis B virus infection, viral modulation of IRF7 stability reduces AIM2 transcription.^215^ However, the host has also evolved strategies to counteract immune evasion. For example, in the context of *Francisella novicida* infection, the interferon-inducible GTPase IRGB10 promotes AIM2 activation by disrupting cytosolic bacteria during infection, thereby releasing microbial dsDNA for sensing.^216^ These examples highlight the sophisticated interplay between host and pathogen to modulate AIM2-mediated immune responses.

Elevated AIM2 expression has also been reported in inflammatory, autoimmune, and metabolic disorders, including psoriasis, systemic lupus erythematosus, chronic kidney disease, diabetes, atherosclerosis, atopic dermatitis, ulcerative colitis, arthritis, and cardiomyopathy, as well as in traumatic and ischemic brain injuries.^217^ Salivary gland epithelial cells from patients with Sjögren’s syndrome show increased AIM2 expression. These cells also exhibit functional activation, which is driven by cytoplasmic DNA accumulation resulting from DNase1 deficiency.^218^ AIM2 has also been implicated in microbe-induced inflammation in oral diseases.^219^

In cancer, the role of AIM2 is complex and context-dependent. AIM2, originally identified as Absent in Melanoma 2 because of its reduced expression in melanoma cells, was initially thought to function as a tumor suppressor.^220,221^ However, subsequent studies have shown that AIM2 can have both pro- and anti-tumor effects, depending on the cancer type and inflammatory context. For example, AIM2 can suppress colorectal cancer by restricting intestinal stem cell proliferation.^222^ In contrast, elevated AIM2 levels have been observed in cutaneous squamous cell carcinoma, HPV-associated cervical cancer, and non-small cell lung cancer.^223^ Additionally, elevated AIM2 expression has been linked to oral squamous cell carcinoma, suggesting that AIM2 may be a potential biomarker in head and neck cancers.^224,225^

Structural studies have shown that upon DNA binding, the HIN domain facilitates AIM2 oligomerization and subsequent recruitment of ASC through PYD–PYD interactions, initiating complex assembly.^196,197,200^ Structure-guided drug design could focus on blocking the DNA-binding groove in the HIN domain to prevent activation or on disrupting the AIM2–ASC interface to inhibit the assembly of this complex and downstream inflammation and cell death. Another strategy may involve stabilizing AIM2 in its autoinhibited conformation to impair oligomerization. Additionally, the mechanisms used by viruses to evade AIM2 activation (such as those involving VP22 from HSV-1 or pUL83 from HCMV) offer a basis for developing peptide- or small molecule-based mimetics to similarly inhibit AIM2 function. Finally, understanding the structural consequences of disease-associated AIM2 mutations could guide the design of modulators.^226^

Beyond informing strategies to inhibit AIM2 inflammasome activation, a structural understanding of the AIM2-PANoptosome could also provide key insights into targeting its formation and subsequent cell death and inflammation. The AIM2-PANoptosome drives PANoptosis independently of NLRP3 and NLRC4 in response to pathogens such as *F. novicida* and HSV-1. AIM2 acts as an upstream regulator in PANoptosis, facilitating cooperative roles for Pyrin and ZBP1 and recruiting additional components to the complex.^24^ The role of AIM2 in immune responses and disease pathogenesis within the context of PANoptosis remains a critical area of ongoing investigation.

Collectively, these insights position AIM2 as a critical sensor that assembles innate immune sensor networks and forms a PANoptosome, making it  a structurally tractable target for therapeutic intervention across a wide spectrum of infectious, autoimmune, and malignant diseases.

## Pyrin

### Structural features of Pyrin

Pyrin acts as a crucial component of the innate immune system, serving as a sensor to detect both sterile and pathogenic triggers, often through mechanisms involving RhoA GTPase inhibition.^12^ The RhoA GTPase is a key regulator of the cytoskeleton and various cellular functions, and its activity can be disrupted by certain bacterial toxins and viral proteins.^227–230^ Such disruptions are known to trigger the activation of Pyrin, which can assemble into an inflammasome in two independent steps.^231,232^ The first step involves dephosphorylation of Pyrin and detachment of the chaperone protein 14-3-3. The second step entails a conformational change in Pyrin facilitated by interaction with the actin cytoskeleton and microtubules, enabling its interaction with the adaptor ASC and the subsequent recruitment of caspase-1 and thereby completing the inflammasome complex.^12,233^

**Fig. 5:**
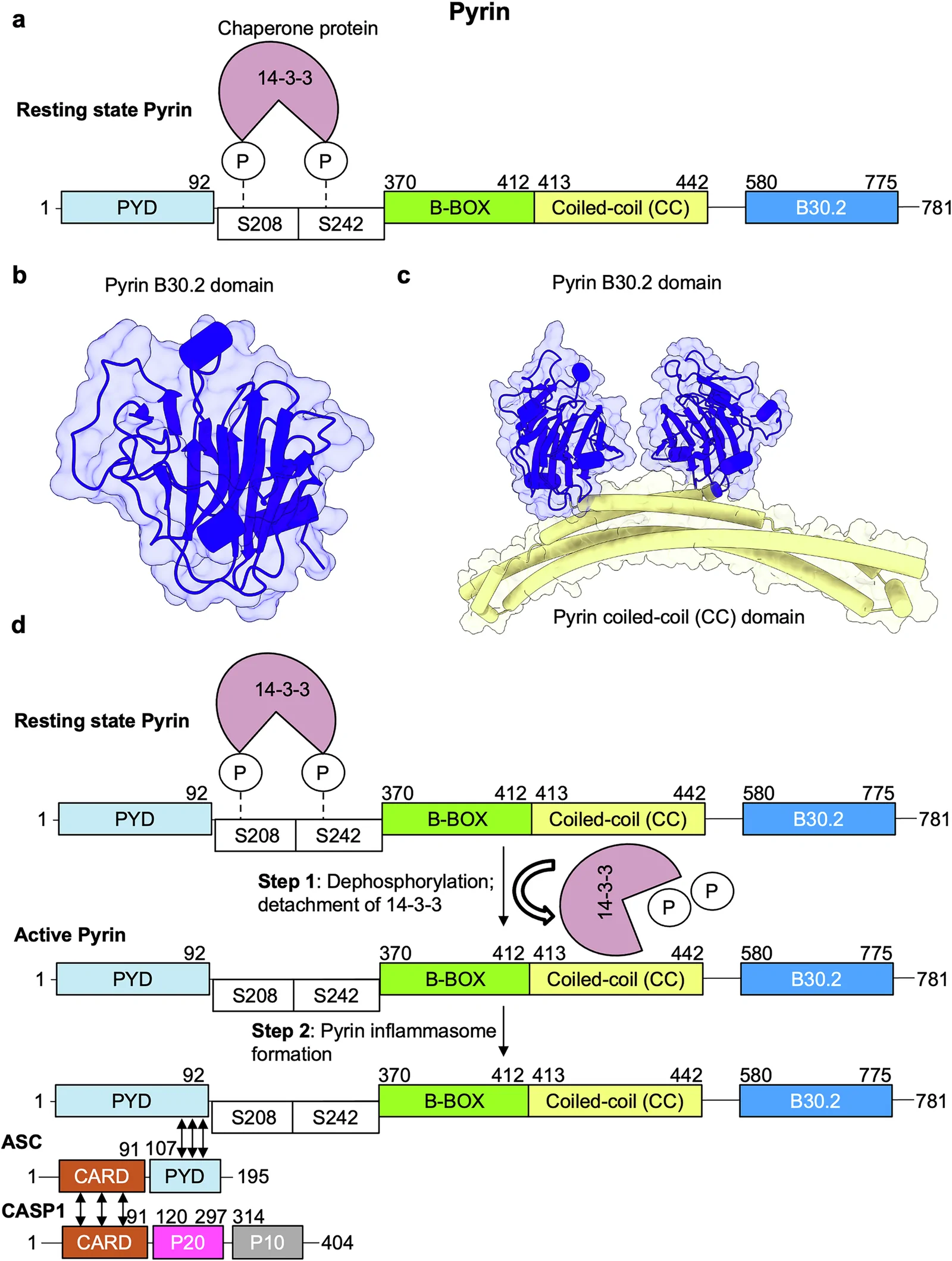
Structural insights into Pyrin activation. **a** Domain architecture of Pyrin. The linker region contains phosphorylation sites that bind the chaperone protein 14-3-3, keeping Pyrin in an inactive state. Residue numbers for domain boundaries are indicated based on the human protein sequence. **b** Crystal structure of the Pyrin B30.2 domain (PDB: 2WL1), showing a β-barrel fold composed of two antiparallel β-sheets forming a compact structure. Prominent surface loops serve as hotspots for FMF-associated mutations. **c** The crystal structure of the CC and B30.2 domains of Pyrin showing the side view of the coat-hanger-like structure (PDB: 4CG4). **d** Schematic representation of the proposed two-step activation model of the Pyrin inflammasome. Step 1: dephosphorylation of Pyrin leads to the detachment of the chaperone protein 14-3-3. Step 2: Pyrin undergoes a conformational change upon exposure to pathogens/triggers. This change exposes the PYD, enabling its interaction with the adaptor protein ASC via homotypic PYD–PYD interactions and resulting in a PYD filament that oligomerizes the CARDs of ASC molecules to mediate caspase-1 recruitment. This results in the subsequent assembly of the Pyrin inflammasome. Domains are color-coded as in **a**.

Although the complete architecture of the Pyrin sensor and its inflammasome remain unresolved, crystallographic, biophysical, and biochemical studies have shed light on key features of its component domains and regulatory mechanisms (Table 2). Pyrin is composed of five distinct domains: a PYD at the N-terminus; a central linker region containing the phosphorylated S208 and S242 residues; a B-box zinc finger domain; a coiled-coil (CC) domain involved in oligomerization; and a C-terminal B30.2 (PRY/SPRY) domain^234,235^ (Fig. 5a).

The crystal structure of the human B30.2 domain shows a compact β-barrel with a jelly-roll topology composed of two antiparallel β-sheets flanked by surface-exposed loops (Fig. 5b). These loops form a positively charged pocket involved in ligand binding and pathogen sensing. Notably, the pocket is enriched with familial Mediterranean fever (FMF)-associated mutations, including M680I, M694V, and V726A.^236^ These mutations are thought to disrupt intramolecular autoinhibitory interactions or alter pathogen recognition, leading to constitutive inflammasome activation and disease pathology.^236^

Further insights into Pyrin assembly come from the crystal structure of a C-terminal fragment encompassing both the CC domain and the B30.2 domain.^237^ This structure resembles a “coat-hanger” architecture, in which the elongated CC region forms a central dimerization scaffold. The B30.2 domains are flexibly tethered to this scaffold via linker loops, allowing dynamic positioning relative to the central axis (Fig. 5c). Each CC monomer folds into four antiparallel α-helices spanning ~155 Å, with a dimerization interface of over 4700 Å². The B-box N-termini are positioned ~170 Å apart, while the B30.2 domains cluster near the two-fold axis of the CC dimer.^237^ Overall, the data from this study suggest that the CC domain forms extended helices that are capable of mediating dimer or oligomer formation, while the adjacent B30.2 domain likely serves as a regulatory sensor.^237^ This structure supports the model that the CC domain enables multimeric assembly and potentially acts as a scaffold for interaction with other inflammasome components. The B30.2 domain acts to regulate this assembly.^237^ Additionally, structural analyses of Pyrin–ASC complexes have highlighted the importance of PYD–PYD interactions in nucleating inflammasome assembly. The Pyrin PYD adopts a canonical six-helix bundle that engages ASC-PYD through homotypic interactions, facilitating ASC polymerization and speck formation. These ASC specks serve as platforms for downstream caspase-1 activation^238^ (Fig. 5d).

In summary, these structural studies converge on a model in which Pyrin activation and inflammasome formation are tightly regulated by a balance among its oligomeric state (driven by the CC domain), its sensing of changes in Rho activity (rather than direct recognition through the B30.2 domain), and its PYD-mediated recruitment of ASC. Mutations, particularly in the B30.2 domain, can destabilize this regulatory balance, leading to the pathological inflammasome activation observed in FMF and other autoinflammatory syndromes.^236,238^ This highlights the importance of Rho signaling in Pyrin inflammasome regulation rather than direct ligand binding.

While these structural features define the capacity of Pyrin for inflammasome assembly, its activation is predominantly regulated by post-translational mechanisms. Under basal conditions, the kinases PKN1 and PKN2 act downstream of the RhoA GTPase to phosphorylate Pyrin at Ser208 and Ser242. These phosphorylation events create binding sites for 14-3-3 proteins, whose binding maintains Pyrin in an autoinhibited conformation (Fig. 5d). During infection, RhoA becomes inactivated, leading to Pyrin dephosphorylation. As a result, 14-3-3 proteins dissociate, and Pyrin then undergoes a conformational change that allows the recruitment of ASC and inflammasome assembly.^231,232^

### Targeting Pyrin and its role in diseases

Although Pyrin has been less extensively reviewed than other sensors, its contributions to autoinflammatory disorders have been discussed in disease-focused literature.^239,240^ While Pyrin plays a protective role in host defense, it is also implicated in a spectrum of autoinflammatory diseases.^241^ Specifically, Pyrin is associated with FMF and various other conditions in which mutations in or dysregulations of Pyrin contribute to excessive inflammation and immune system activation. FMF, primarily caused by mutations in the *MEFV* gene that encodes Pyrin, often involves alterations in the C-terminal B30.2 domain of Pyrin.^242^ Mutations such as M680I, E148Q, and V726A are associated with milder forms of FMF, whereas the M694V mutation is linked to more severe disease manifestations.^243^

Pyrin dysregulation also contributes to other periodic fever syndromes, including Hyper-IgD syndrome (HIDS) and pyogenic arthritis, pyoderma gangrenosum, and acne (PAPA) syndrome.^229,244^ In PAPA syndrome, mutations in PSTPIP1, a Pyrin-interacting protein, enhance PSTPIP1 phosphorylation and its binding affinity for Pyrin, thereby promoting inflammasome assembly and chronic inflammation.^245^

Given the extensive connections between Pyrin dysregulation and inflammatory diseases, therapeutic strategies targeting Pyrin-mediated inflammation have been investigated, with colchicine being the most widely used therapeutic agent for FMF.^246,247^ Colchicine inhibits microtubule polymerization, indirectly suppressing Pyrin activation.^248^ However, it is not a specific inhibitor and also inhibits NLRP3, which likely contributes to its clinical efficacy in broader inflammatory conditions.^249–251^ Despite its widespread use, the nonspecific mechanism of colchicine highlights the need for more targeted therapies to modulate Pyrin-mediated immune responses without affecting other innate immune sensors.

Given that Pyrin is relatively understudied compared to other sensors, further research into its activation pathways, domain dynamics, and crosstalk with other sensors is needed. Future studies should prioritize the structural and biochemical characterization of the full-length Pyrin protein to determine its regulatory mechanisms and its precise role in inflammasome activation. Advanced techniques such as cryo-ET could be employed to visualize Pyrin’s native conformations and its assembly into inflammasomes, providing insights into its dynamic structural transitions during activation. Additionally, the interactions of Pyrin with kinases such as PKN1/2, which facilitate its binding to the chaperone protein 14-3-3 to maintain Pyrin in an inactive state, warrant further elucidation. Understanding how these kinases regulate Pyrin activity could identify new therapeutic targets for Pyrin-mediated inflammation. Furthermore, efforts should be directed toward developing specific Pyrin inhibitors, which could provide more precise therapeutic options than nonspecific treatments like colchicine. Collectively, these research approaches could offer new treatment strategies for patients with Pyrin-mediated immune dysregulation and inflammatory conditions.

## Other molecules suggested to form inflammasomes and panoptosomes

In addition to NLRP1, NLRP3, NLRC4, AIM2, and Pyrin, several other cytosolic innate immune sensors have been suggested to form inflammasomes and PANoptosomes in specific cell types or in context-dependent manners. Limited structural information is available for many of these sensors (Table 2), which has complicated our ability to understand their activation mechanisms and complex formation. Therefore, this section focuses on genetic, biochemical, imaging, and functional studies of these sensors. Continued research is necessary to provide structural insights that will clarify their roles, mechanisms of activation, and potential contributions to inflammation and immunity.

### NLRC5

NLRC5 is a member of the NLR family that is highly expressed in immune cells and tissues associated with mucosal surfaces. It was originally identified as a key transcriptional regulator of major histocompatibility complex class I (MHC-I) genes, a function that involves nuclear shuttling.^252^ NLRC5 has been implicated in broader immunoregulatory functions, including modulation of NF-κB and type I IFN signaling pathways.^253,254^ Structurally, human NLRC5 contains an atypical CARD at the N-terminus and an LRR domain at the C-terminus.^255^

In the context of inflammasomes, a co-immunoprecipitation study showed that NLRC5 interacts with NLRP3 and ASC, promoting the production of IL-1β in certain cellular contexts.^256^ More recent studies have provided genetic, biochemical, and molecular evidence showing that NLRC5 forms a PANoptosome, with the NLRP3 inflammasome as an integral component, to drive PANoptosis in response to NAD^+^ depletion, which occurs in infections, hemolytic diseases, and other inflammatory conditions^28^ (Table 2). Although the structural basis of this interaction is still unknown, genetic and molecular analyses show that the NLRC5-PANoptosome plays a key physiological role in promoting pro-inflammatory cytokine production and inflammation.^28^

Together, these molecular studies suggest that NLRC5 does not directly form an inflammasome, but that it is critical for incorporating the NLRP3 inflammasome into a larger PANoptosome complex to drive cell death and inflammation. These recent insights highlight NLRC5 as a multifunctional regulator of immune signaling, with roles that extend beyond MHC-I transcription to include the regulation of cell death and inflammation. Therefore, NLRC5 represents a potential target for therapeutic investigation in inflammatory and infectious diseases.

### NLRP12

NLRP12 was initially identified and characterized in the HL60 human leukemia cell line.^257,258^ It is composed of a PYD at the N-terminus, a central NBD, and an LRR domain at the C-terminus. NLRP12 is primarily expressed in myeloid cells, such as neutrophils, eosinophils, monocytes, macrophages, and immature dendritic cells.^259^

NLRP12 acts as a negative regulator of inflammation by binding to and inhibiting phosphorylated IRAK1, thereby suppressing Toll-like receptor (TLR) signaling and modulating immune responses.^260^ NLRP12 expression is upregulated following TLR activation through the binding of B lymphocyte-induced maturation protein-1 to its promoter region.^261^ Additionally, NLRP12 undergoes alternative splicing, giving rise to several transcript variants, although the functional significance of these isoforms remains poorly understood.^259^

Beyond its anti-inflammatory role, NLRP12 modulates cytokine production in specific infectious contexts. During infection with *Yersinia pestis* or *Plasmodium chabaudi*, NLRP12 induces the release of IL-1β and IL-18, contributing to host protection against these pathogens.^262,263^ By contrast, in infections caused by *Salmonella* Typhimurium, *Klebsiella pneumoniae*, or *Mycobacterium tuberculosis*, as well as in models of colonic tumorigenesis, NLRP12 can suppress NF-κB and MAPK signaling pathways, thereby limiting the production of pro-inflammatory and tumor-promoting mediators.^260,264^ Consistent with this, NLRP12 deficiency results in enhanced ERK and NF-κB activation. This enhanced signaling upregulates mediators such as CXCL13 and AKT, thereby exacerbating colitis and colorectal cancer.^265,266^ NLRP12 also functions as an intrinsic regulator of T cells, restraining IL-4 production and NF-κB signaling and thereby limiting T cell-driven inflammation in models of colitis, dermatitis, and neuroinflammation.^267^

Although NLRP12 was initially proposed to form an inflammasome complex, more recent genetic and biochemical studies indicate that it functions as part of a broader NLR network, assembling a PANoptosome complex with the NLRP3 inflammasome as an integral component^27,268^ (Table 2). In this context, biochemical and imaging studies show that NLRP12 interacts with NLRP3 and NLRC5 to form a PANoptosome, contributing to both inflammasome-dependent cytokine production and lytic cell death in response to triggers such as heme and microbial ligands.^27^ Within the PANoptosome, NLRP12 and NLRP3 have overlapping roles in inflammasome activation, with both contributing to inflammasome-dependent cytokine production, although NLRP3 is dispensable for cell death, while NLRP12 is required.^27,28^

Overall, these findings highlight the context-dependent roles of NLRP12 in both restraining and promoting inflammation. Its dual functions as a suppressor of pro-inflammatory signaling and a driver of inflammasome activation, PANoptosome formation, and PANoptosis make it an attractive but complex therapeutic target. These context-dependent functions may be influenced by cell- or tissue-specific expression levels of NLRP12, as suggested by studies in colorectal cancer models, although further investigation is needed. Elucidating the molecular determinants governing NLRP12 activity will facilitate the identification of strategies for therapeutic modulation in inflammatory and infectious diseases.

### NLRP6

NLRP6 was initially identified in the human cell lines COS-7L and 293T, where it was shown to induce caspase-1 and NF-κB activation, suggesting a role as a sensor capable of assembling inflammasome complexes.^269^ NLRP6 is characterized by its NBD and LRR domains. These structural features suggest that it can function as a cytosolic sensor in innate immunity.

Functionally, NLRP6 is proposed to detect bacterial components through its LRR domain. One study showed that it can detect lipoteichoic acid from gram-positive bacteria and LPS from gram-negative bacteria, triggering conformational changes that lead to its oligomerization.^270^ This study suggested that, upon activation, NLRP6 assembles with ASC and pro-caspase-1 to form an inflammasome complex, resulting in the cleavage and activation of the pro-inflammatory cytokines IL-1β and IL-18.^270^

In addition to recognizing bacterial molecules, recent studies have shown that NLRP6 can interact with double-stranded RNA (dsRNA) and other ligands. This interaction, facilitated by a poly-lysine sequence (K350–354), induces LLPS, a process essential for efficient inflammasome activation and recruitment of downstream signaling molecules, thereby regulating NLRP6 activity.^271^ The ability of NLRP6 to respond to both bacterial and viral components suggests that it may have versatile roles in anti-microbial immunity against infections,^271,272^ though additional work is needed to fully elucidate its functions.

Studies have also suggested that NLRP6 has roles in maintaining intestinal barrier function and regulating gut microbiota composition. Mice deficient in NLRP6 exhibit reduced systemic IL-18 levels and altered colonic epithelial responses.^273^ These mice display significant dysbiosis and altered microbial diversity, which can exacerbate or protect against disease in a context-dependent manner. For instance, NLRP6-deficient mice have shown increased resistance to bacterial infections caused by *L. monocytogenes*, *Salmonella* Typhimurium, or *Escherichia coli*.^273^ Further research is needed to fully understand the mechanisms through which NLRP6 influences gut microbiota and inflammation to identify new therapeutic targets for managing inflammatory diseases, such as inflammatory bowel disease and colorectal cancer.

### NLRP9

NLRP9 is another member of the NOD-like receptor family. The human genome encodes a single *NLRP9* gene, whereas mice possess three paralogs (*Nlrp9a*, *Nlrp9b*, and *Nlrp9c*), highlighting potential species-specific diversification.^274–276^ Like other NLRP family members, NLRP9 contains an N-terminal PYD, a central NACHT domain with ATPase activity, and C-terminal LRRs.^274,275^ In humans, NLRP9 is highly expressed in oocytes and clusters phylogenetically with development-associated NLRs, suggesting possible roles beyond innate immunity.^274,276^ NLRP9 is also enriched in intestinal epithelial cells, suggesting key roles in mucosal immunity,^275^ although its functions in other cell types require further study. In mice, the NLRP9b isoform interacts with the RNA helicase DHX9 in response to rotavirus-derived dsRNA, contributing to viral clearance.^275^ However, the extent to which this pathway reflects inflammasome activity remains under investigation, and its physiological significance is not yet fully understood.

Structural insights into NLRP9 remain limited.^277–279^ Crystal structures of the human NLRP9 PYD identified a canonical death-domain fold with a distinctive bent N-terminal loop. The shape of this loop differs from those observed in ASC,^280^ AIM2,^196,200^ and other PYD-containing sensors.^91,92^ This unique conformational feature has been proposed to modulate PYD-mediated homotypic interactions and may influence adaptor engagement or filament assembly.^277,278^ How the PYD-dependent signaling and NACHT-driven oligomerization are structurally coupled to the DHX9-mediated RNA sensing observed in mice remains unresolved. Further studies are needed to clarify the potential functions of NLRP9 in infectious and inflammatory diseases.

### IFI16

IFI16 and AIM2 are members of the AIM2-like receptor (ALR) family, and they share a conserved domain organization consisting of an N-terminal PYD and two C-terminal HIN200 domains that mediate dsDNA binding.^196,197,281–283^ However, IFI16 is encoded in humans but not in mice, limiting the use of murine models to study its function.^282,283^ Furthermore, despite their shared architecture, IFI16 and AIM2 differ markedly in their structural organization. AIM2 contains a single HIN domain and directly assembles into multi-protein complexes upon cytosolic DNA binding by promoting PYD-mediated ASC polymerization.^196,197^ In contrast, IFI16 contains two HIN domains (HIN-A and HIN-B), allowing it to engage dsDNA through multiple binding modes and configurations rather than a single binding interface as seen in AIM2.^281,283^ Structural studies indicate that IFI16 recognizes dsDNA in a sequence-independent manner through electrostatic interactions between positively charged surfaces of the HIN domains and the DNA sugar-phosphate backbone.^281,284^ Although the HIN domains mediate DNA recognition, they do not independently promote oligomerization. Instead, PYD-driven homotypic interactions facilitate higher-order filament formation and downstream signaling.^285^ Collectively, these features suggest that IFI16 functions more as a context-dependent DNA sensor rather than as a scaffold for multi-protein complex formation.

Functionally, IFI16 has been implicated in inflammasome formation and cell death. During infection with Kaposi sarcoma-associated herpesvirus, IFI16 translocates from the nucleus to the cytoplasm, where it interacts with the adaptor ASC and pro-caspase-1 to form a functional inflammasome complex.^286^ Beyond its role in inflammasome signaling, IFI16 has been implicated in promoting cell death during incomplete HIV infection, contributing to HIV-associated immune depletion.^287^

The ability of IFI16 to discriminate between foreign DNA within the nucleus and host DNA may depend on specific DNA structural determinants. However, the exact mechanisms that coordinate IFI16 with other DNA sensors remain unclear. Moreover, emerging evidence suggests that IFI16 may coordinate with AIM2 in DNA sensing and inflammasome assembly.^288^ This potential interaction represents a key area for future research to understand how these sensors work together in response to viral infections or DNA damage. Further studies into IFI16’s structural determinants, regulatory mechanisms, and interactions with AIM2 and other DNA sensors will be critical for advancing therapeutic strategies for viral infections, autoimmunity, and chronic inflammatory diseases.

## Summary and future perspectives

Beyond the foundational understanding gained from genetic, biochemical, and molecular imaging studies, recent advances in structural biology, particularly cryo-EM, have substantially overcome prior experimental limitations posed by the aggregation-prone nature of cytosolic innate immune sensors. These breakthroughs have defined both shared and sensor-specific mechanisms of activation among the innate immune sensors that form inflammasomes and PANoptosomes. These sensors have different domain architectures and upstream triggers. Yet all undergo conformational transitions from autoinhibited to active states. This enables filamentous assembly through PYD–PYD, CARD–CARD, or heterotypic interactions to form inflammasomes and PANoptosomes. Insights from cryo-EM, cryo-ET, crystallography, and biochemical analyses have been instrumental in defining these mechanisms and their therapeutic implications.

Translationally, it is now broadly recognized that targeting innate immune sensors and their downstream inflammasome and PANoptosome pathways provides therapeutic strategies across the disease spectrum. Emerging structural studies distinguish therapeutic strategies that regulate complex assembly from those that target downstream execution. Several agents that directly target sensors to block complex assembly have demonstrated preclinical efficacy,^154^ and some have progressed to clinical trials.^86,145,148,149^ These successes suggest that upstream targeting may be the most clinically efficacious strategy. When targeting at the sensor level is not possible, downstream targeting provides an alternative way to reduce inflammatory outputs. Caspase-1 inhibition can reduce IL-1β and IL-18 maturation.^154,289^ Additionally, inhibiting GSDMD prevents pore formation and membrane disruption, allowing inflammatory signaling to be reduced without completely abolishing upstream innate immune sensing.^154^ Beyond these strategies, membrane-proximal regulatory steps are emerging as additional therapeutic opportunities.^290–292^ Innate immune sensor activation has been linked to membrane composition, lipid organization, and subcellular localization,^87,94–96^ and the disruption of lipid–protein interactions can limit sensor clustering and complex assembly.^293,294^ These strategies remain exploratory but are conceptually supported by structural and cellular biophysical studies.

Combination therapy may also be beneficial. For instance, concurrent removal of RIPK3 and caspase-1-mediated cytokine maturation suppresses tissue injury more effectively than either strategy alone,^35^ suggesting key roles for combination therapies in blocking PANoptosis in tissue damage and disease.

Biomarker-guided selection may further improve treatment specificity. Although markers such as phospho-RIPK3, cleaved gasdermins, and activated caspase-8 have been widely used in preclinical experimental settings, their use in routine clinical diagnostics remains limited.^24,27,35^ In clinical contexts, IL-1β, IL-18, C-reactive protein, and ferritin have the potential to serve as indicators of inflammasome and PANoptosome activity to inform treatments.^295,296^ As insight into inflammasome and PANoptosome regulation deepens, the integration of clinically tractable biomarkers with mechanism-based therapeutic strategies will become increasingly critical. Ultimately, this strategy has the potential to enhance efficacy while limiting off-target or excessive immune suppression.

### Open questions

Despite several recent advances, many key questions remain unanswered in inflammasome and PANoptosome research. Broadly, these include the identification of additional physiologically relevant triggers, upstream regulatory processes, complex-forming innate immune sensors, complex components, and downstream executioners.

At the sensor level, open areas of research include understanding the mechanisms of ligand recognition by the sensors and how this interaction leads to the recruitment of other molecules to form complexes and activate signaling pathways. Addressing these knowledge gaps will require further structural studies using cutting-edge technologies such as cryo-ET along with complementary functional studies, including live-cell imaging, quantitative proteomics, gene editing, and biochemical assays. These integrative approaches will be instrumental in dissecting the overall activation of innate immune sensors, as well as their protein–protein interactions, PTMs, and cellular signaling pathways, in real time. Given the critical role of these sensors and their inflammasome and PANoptosome complexes in disease, these studies may pave the way for the identification of new therapeutic targets.

Furthermore, to date, structural investigations of inflammasome assembly have used individual ligands or triggers, which oversimplifies the physiological environment encountered in the body, where multiple stimuli are present simultaneously. Under more physiological conditions, genetic and biochemical evidence shows that inflammasomes frequently integrate into PANoptosomes. Full atomic-resolution structures of both inflammasomes and PANoptosomes remain unavailable, and future studies will be needed to define them. While both inflammasomes and PANoptosomes are conclusively established through genetic, biochemical, imaging, and functional analyses,^24–38^ challenges remain in defining how individual sensors interface with core complex components such as caspase-8, ASC, RIPKs, and others. Additionally, the stoichiometry, architecture, and assembly dynamics of these complexes are not well defined. Moreover, additional molecules may be present in PANoptosomes that remain to be identified. Addressing these questions will require integrated structural, biophysical, and quantitative cell-based approaches to resolve inflammasome and PANoptosome composition and dynamics at high resolution, which will be essential for translational applications. From a therapeutic perspective, strategies are likely to be most effective if they target individual sensors, such as NLRP1, rather than shared components like ASC or caspase-1. This approach could achieve pathway specificity while minimizing broad suppression of innate immunity. Small-molecule inhibitors for certain sensors remain under development and in clinical trials, while therapeutic targeting of PANoptosome complexes as a whole remains at the preclinical stage. Gaining further structural insights into these molecules will enable this type of targeted application.

Overall, continued advances in structural immunology and functional interrogation of innate immune sensor biology are essential for deepening our mechanistic understanding of these molecules. Integrating inflammasome and PANoptosome research will enhance our knowledge of their physiological relevance and critically inform the development of novel, structure-guided therapeutic strategies to modulate the activity of innate immune sensors in inflammation and disease. These efforts hold promise for addressing a range of autoinflammatory, infectious, and immune-related diseases and cancers.
